# RETRACTION: Human‐in‐the‐Loop Artificial Intelligence System for Systematic Literature Review: Methods and Validations for the AutoLit Review Software

**DOI:** 10.1002/cesm.70086

**Published:** 2026-06-01

## Abstract

**RETRACTION:** K.M. Kallmes, J. Thurnham, M. Sauca, R. Tarchand, K.R. Kallmes, and K.J. Holub, “Human‐in‐the‐Loop Artificial Intelligence System for Systematic Literature Review: Methods and Validations for the AutoLit Review Software,” *Cochrane Evidence Synthesis and Methods* 3, no. 6 (2025): e70059, https://doi.org/10.1002/cesm.70059.

The above article, published online on 25 October 2025 in Wiley Online Library (http://onlinelibrary.wiley.com/), has been retracted by agreement among the authors; Cochrane's Deputy Editor‐in‐Chief, Toby Lasserson; the Cochrane Research Integrity team; and John Wiley & Sons Ltd., due to noncompliance with Cochrane's Conflict of Interest (COI) policy as it relates to Cochrane content.

At submission, the authors disclosed that they were all currently employed by Nested Knowledge, a commercial entity whose product is evaluated in the article, and that all authors hold equity in the company. These relationships constitute a breach of Cochrane's COI policy. Due to an editorial oversight, this policy breach was not identified during the editorial process, leading to the article's acceptance and publication. However, as the COIs are in breach of Cochrane's COI policy, the article must be retracted.

This article was an invited submission, and the editors were aware that the authors were the developers of the tool described. The decision to retract reflects Cochrane's policies on conflicts of interest and does not relate to any failure by the authors to disclose such commercial interests, concerns over research ethics, or issues with the content or integrity of the article. The handling editor accepts responsibility for the oversight.

**RETRACTION:** K.M. Kallmes, J. Thurnham, M. Sauca, R. Tarchand, K.R. Kallmes, and K.J. Holub, “Human‐in‐the‐Loop Artificial Intelligence System for Systematic Literature Review: Methods and Validations for the AutoLit Review Software,” *Cochrane Evidence Synthesis and Methods* 3, no. 6 (2025): e70059, https://doi.org/10.1002/cesm.70059.

The above article, published online on 25 October 2025 in Wiley Online Library (http://onlinelibrary.wiley.com/), has been retracted by agreement among the authors; Cochrane's Deputy Editor‐in‐Chief, Toby Lasserson; the Cochrane Research Integrity team; and John Wiley & Sons Ltd., due to noncompliance with Cochrane's Conflict of Interest (COI) policy as it relates to Cochrane content.

At submission, the authors disclosed that they were all currently employed by Nested Knowledge, a commercial entity whose product is evaluated in the article, and that all authors hold equity in the company. These relationships constitute a breach of Cochrane's COI policy. Due to an editorial oversight, this policy breach was not identified during the editorial process, leading to the article's acceptance and publication. However, as the COIs are in breach of Cochrane's COI policy, the article must be retracted.

This article was an invited submission, and the editors were aware that the authors were the developers of the tool described. The decision to retract reflects Cochrane's policies on conflicts of interest and does not relate to any failure by the authors to disclose such commercial interests, concerns over research ethics, or issues with the content or integrity of the article. The handling editor accepts responsibility for the oversight.

